# Mapping of the New Fertility Restorer Gene *Rf-PET2* Close to *Rf1* on Linkage Group 13 in Sunflower (*Helianthus annuus* L.)

**DOI:** 10.3390/genes11030269

**Published:** 2020-03-01

**Authors:** Osama Sajer, Uta Schirmak, Sonia Hamrit, Renate Horn

**Affiliations:** Institut für Biowissenschaften, Pflanzengenetik, Universität Rostock, Albert-Einstein-Str. 3, 18051 Rostock, Germany; osamasajer@hotmail.com (O.S.); uta-schirmak@web.de (U.S.); s.hamrit@strube-research.net (S.H.)

**Keywords:** *Rf-PET2*, *Rf1*, fertility restoration, cytoplasmic male sterility (CMS), sunflower, *Helianthus*, marker, hybrid breeding, new CMS sources

## Abstract

The PET2-cytoplasm represents a well characterized new source of cytoplasmic male sterility (CMS) in sunflower. It is distinct from the PET1-cytoplasm, used worldwide for commercial hybrid breeding, although it was, as PET1, derived from an interspecific cross between *Helianthus. petiolaris* and *H. annuus*. Fertility restoration is essential for the use of CMS PET2 in sunflower hybrid breeding. Markers closely linked to the fertility restorer gene are needed to build up a pool of restorer lines. Fertility-restored F_1_-hybrids RHA 265(PET2) × IH-51 showed pollen viability of 98.2% ± 1.2, indicating a sporophytic mode of fertility restoration. Segregation analyses in the F_2_-population of the cross RHA 265(PET2) × IH-51 revealed that this cross segregated for one major restorer gene *Rf-PET2*. Bulked-segregant analyses investigating 256 amplified fragment length polymorphism (AFLP) primer combinations revealed a high degree of polymorphism in this cross. Using a subset of 24 AFLP markers, three sequence-tagged site (STS) markers and three microsatellite markers, *Rf-PET2* could be mapped to the distal region of linkage group 13 between ORS1030 and ORS630. Three AFLP markers linked to *Rf-PET2* were cloned and sequenced. Homology search against the sunflower genome sequence of HanXRQ v1r1 confirmed the physical location of *Rf-PET2* close to the restorer gene *Rf1* for CMS PET1. STS markers were mapped that can now be used for marker-assisted selection.

## 1. Introduction

Cytoplasmic male sterility (CMS) is a maternally inherited trait in higher plants, in which these plants fail to develop or shed functional pollen. Rearrangements in the mitochondrial genome correlated with CMS have been identified for several CMS sources [1,2]. Fertility restoration by dominant nuclear restorer genes is essential to produce fully fertile F_1_-hybrids that allow exploitation of CMS/Rf-systems for commercial hybrid breeding [3].

In sunflower, only one CMS system, the so called PET1-cytoplasm, has so far been used for commercial hybrid breeding [4], although more than 70 CMS sources have been described [5]. Up to now, the lack of restorer lines and molecular markers closely linked to the restorer genes has hampered the use of the new CMS sources as well as the development of new restorer and maintainer pools for these cytoplasms. However, using only one CMS cytoplasm, as demonstrated by the T-cytoplasm in maize, carries a high risk of pathogens specializing on this cytoplasm, which can lead to heavy yield losses [6]. Therefore, an interest exists for introducing new CMS cytoplasms into commercial sunflower breeding.

The CMS PET2 (also known as CMG-1), which originated from an interspecific cross between *Helianthus petiolaris* and *H. annuus* [7], is one of the new CMS sources that is distinct from the PET1 cytoplasm [8], although both CMS cytoplasms were derived from the same interspecific cross. Molecular analyses demonstrated that the PET2 cytoplasm does not contain any homology to *orfH522* or the typical mitochondrial rearrangements of the PET1 cytoplasm [9,10,11]. Regarding the diversity between 28 CMS sources and the fertile cytoplasm, the hybridization patterns of CMS PET2 differed from the fertile cytoplasm for the mitochondrial genes *atp6*, *atp9*, *cob*, and *orfH708* [11]. These differences on mitochondrial DNA level were shared with CMS GIG1, which originated from an interspecific cross of *H. giganteus* with *H. annuus* [12]. CMS PET2 and CMS GIG1 were also both characterized by the expression of an additional mitochondrial encoded 12.4-kDa-protein [13] instead of the 16-kDa-protein typical for CMS PET1 [14], which was shown to be cytotoxic in *Escherichia. coli* [15] and to produce male-sterile transgenic tobacco [16].

The molecular mechanism behind cytoplasmic male sterility in the presence of CMS PET2 has recently been discovered [17]. Two new open reading frames (orfs), *orf288* and *orf231*, which are co-transcribed have been identified as the cause. Due to a duplication of *atp9* followed by an insertion of 271 bp of unknown origin, these two new orfs were created. While *orf288* shares the first 53 bp with *atp9*, *orf231* consists of the last 228 bp of the duplicated *atp9*. The start codon of *orf231* is formed by the 271-bp insertion. In fertility-restored hybrids the co-transcript of *orf288* and *orf231* is reduced, indicating a role in the development of male sterility. The CMS mechanism for CMS PET2 has been confirmed by whole genome sequencing of the mitochondrial DNA [18]. The presence of *orf288* and *orf231* could be also confirmed for CMS GIG1 [17]. In addition, mitochondrial markers have been developed, distinguishing CMS PET1 and CMS PET2. Changes involving the *atp9* gene have also been identified as cause for male sterility in CMS PEF1 in sunflower derived from *H. petiolaris* ssp. *fallax* × *H. annuus* [19]. In this case, a 507-bp insertion adjacent to the 3′-end of the *atp9* gene resulted in transcript changes between fertile and male sterile lines [20].

To identify restorer lines for new CMS sources, test crosses between nine CMS sources (ANL1, ANL2, MAX1, PEF1, PET2, ANN1, ANN2, ANN3, and ANN4) and restorer and maintainer lines of the PET1 cytoplasm were performed. One restorer line of the PET1 cytoplasm, IH-51, resulted in fully restored male fertility in F_1_-hybrids carrying the PET2-cytoplasm [21]. However, another restorer line of the PET1 cytoplasm, RHA 265, represents a maintainer line for CMS PET2. Up to now, only Havekes et al. [22] had also identified a line, RHA 294, fully restoring hybrids carrying CMS PET2 or CMS GIG1, whereas the lines RPET2 and RGIG1 only partially restored male fertility in F_1_-hybrids of these CMS sources. The results clearly showed that CMS PET2 is different from CMS PET1 and could represent an interesting alternative for commercial hybrid breeding.

In sunflower, the restorer genes for CMS PET1 have been extensively analyzed due to the use in commercial hybrid breeding. The restorer gene *Rf1* was first identified using the line T66006-2-1-B in breeding programs [23]. In developing a restorer pool for CMS PET1, *Rf1* has been introduced into several restorer lines like RHA 271, RHA 272, RHA 273, and others by the United States Department of Agriculture Agricultural Research Service [5,24]. In addition, a second dominant fertility restorer gene *Rf2* was observed in an allelic test cross between T66006-2-1-B and MZ01398. However, *Rf2* seems to be present in nearly all inbred lines [5]. *Rf1* is mainly used for restoring pollen fertility in sunflower hybrids [25]. Recently, a third fertility restorer gene *Rf3*, which could be shown to be different from *Rf1* and *Rf2*, was identified in the restorer lines RHA 280 and RHA 340 used for confectionary sunflowers [26,27]. *Rf3* was mapped between simple sequence repeat (SSR) markers ORS966 and ORS328 on linkage group (LG) 7 [28]. In addition, a restorer gene *Rf5* coming from a wild *H. annuus* has recently been mapped near the *Rf1* gene on LG13 [29].

The restorer gene *Rf1* was first assigned to LG 6 in the restriction length polymorphism (RFLP) map of Gentzbittel et al. [30,31], then to LG 2 in the RFLP map by Jan et al. [32], and finally to LG13 of the sunflower reference map [33] using the simple sequence repeat (SSR) marker ORS1030 [34]. The linkage map around the *Rf1* gene consisted of 35 amplified fragment length polymorphism (AFLP) markers, 7 random amplified polymorphic DNA (RAPD) markers, and ORS1030 [34]. Using sequences of cloned markers and bacterial artificial chromosome (BAC) clones hybridizing to these markers, two regions in the sunflower genome sequence HanXRQ v1r1 [35] could be identified as potential physical locations of the *Rf1* gene on LG13 [36]. In the 30-Mb region and the 3.9-Mb region nine potential candidate genes for *Rf1* were annotated: seven pentatricopeptide repeat (PPR) genes, one gene for a poly(A) polymerase 3 (PAPS3), and one for an aldehyde dehydrogenase 21A. Association studies combining next generation sequencing with a candidate gene approach allowed the identification of 10 single nucleotide polymorphisms (SNPs) significantly associated with fertility restoration. These SNPs narrowed the potential candidates for *Rf1* down to three genes, PPR841, PPR861, and PPR621. All three genes are based in the 3.9-Mb region between 169,655,088 (ORS1030) and 173,581,392 (OP-H13) [36]. Genome-wide association studies using whole sunflower genome sequences [37,38,39] and data from SNP arrays [40] had also identified the distal region of LG13 for *Rf1* but could not tag a specific gene due to the insufficient resolution of the whole genome analyses.

Regarding restorer-of-fertility genes of new CMS sources, Feng and Jan [41] mapped an additional restorer gene *Rf4* to LG3 of the sunflower general reference map [33]. *Rf4* originating from *H. maximiliani* restored pollen fertility in the presence of the newly identified CMS GIG2, resulting from an interspecific cross of *H. giganteus × H. annuus* [41]. The restorer gene *Rf6* from *H. angustifolius* was also mapped on LG3. *Rf6* restored fertility to CMS 514A, a male sterile line based on a *H. tuberosus* cytoplasm [42]. These works show that wild sunflower species represent an interesting source for new restorer genes. In the cross RHA 265(PEF1) × LC1064, Schnabel et al. [43] identified AFLP markers linked to the restorer gene *Rf-PEF1*, which represents a major restorer gene for CMS PEF1.

This is the first report of mapping the restorer gene *Rf-PET2*, which fully restores F_1_-hybrids carrying CMS PET2. AFLP markers in combination with SSR markers allowed the assignment of *Rf-PET2* to the distal region of LG13. Comparative mapping of the two restorer genes *Rf-PET2* and *Rf1* using known and newly developed sequence-tagged markers demonstrated that both genes are located adjacent to each other within the PPR gene cluster on LG13. Knowledge about the location of the restorer gene will help to develop a restorer pool for CMS PET2.

## 2. Material and Methods

### 2.1. Plant Material and Field Trials

F2- and F3-populations were derived from the cross RHA 265(PET2) × IH-51. The CMS-line PET2 was maintained by the line RHA 265, which is a restorer line of the PET1-cytoplasm. IH-51 is a restorer line of CMS PET2 as well as of CMS PET1 [21]. F_2_- and F_3_-populations were grown in the field of Groß-Gerau near Frankfurt/Main over several years and evaluated for male fertility/sterility. Leaf material from F_2_-individuals for DNA analyses was immediately frozen in liquid nitrogen and stored at −20 °C. The segregating F_2_-population used for mapping markers to the restorer gene *Rf-PET2* consisted of 199 F_2_-individuals. For mapping of the *Rf1* gene, F_2_- and F_3_-populations were derived from the cross RHA 325(PET1) × HA 342, as described in Horn et al. [44], and enlarged to 183 F_2_-individuals.

### 2.2. Staining for Pollen Viability

Pollen viability was assessed for 10 fertile plants of RHA 265(PET2) × IH-51 hybrids by Alexander’s staining [45]. For each plant, two separate samples of 100 pollen grains were counted. Pollen viability was also estimated for the white pollen inside the medium-sized anthers of the male-sterile plants, which do not shed these pollen grains.

### 2.3. Isolation of Genomic DNA

Genomic DNA was isolated according to the procedure of Doyle and Doyle [46]. In liquid nitrogen, grounded powder of 2.5 g leaf material was incubated with 15 mL extraction buffer (100 mM Tris/HCl pH 8.0, 1.4 M NaCl, 20 mM EDTA, 2% CTAB, 1% Na_2_S_2_O_3_) at 65 °C for 30 min. After chloroform extraction the aqueous phase was obtained by centrifugation. The procedure was repeated and finally the DNA was precipitated in the aqueous phase by adding 1 mL ammonium acetate (10 M) and 1 mL sodium acetate (3 M pH 5.5) in addition to two-thirds volume 2-propanol at 4 °C. High molecular weight DNA was transferred by a glass hook to a new tube and washed once with wash alcohol (70% ethanol, 10 mM ammonium acetate). DNA was briefly dried and then solubilized in 1 mL TE (10 mM Tris/HCl Ph = 8.0, 1 mM EDTA).

### 2.4. AFLP-Analyses

Sunflower genomic DNA was digested with *Eco*RI and *Mse*I and ligated to *Eco*RI and *Mse*I adapters, as described by Vos et al. [47]. For the *Rf-PET2* gene, AFLP analyses were performed based on the preamplification with E01 (5′-GACTGCGTACCAATTCA-3′) and M02 (5′-GATGAGTCCTGAGTAAC-3′) as primers. For the selective amplification, 16 *Eco*RI primers (E31 to E46) and 16 *Mse*I primers (M47 to M62) were combined according to Vos et al. [47]. For the *Rf1* gene, the preamplifications were done using E02 (5′-GACTGCGTACCAATTCC-3′) and M02 (5′-GATGAGTCCTGAGTAAC-3′). For the selective amplification, 16 *Eco*RI primers (E47-E62) were combined with 16 M-Primers (M47-M62). All primer sequences and numbers were used according to Keygene, N.V., Wageningen, NL (http://wheat.pw.usda.gov/ggpages/keygeneAFLPs.html). *Eco*RI primers (labeled with IRD700 or IRD800) from Eurofins MWG Operon (Ebersberg, Germany) were used for non-radioactive labeling of the selective amplification products. PCR products were run on denaturing polyacrylamide gels on a DNA Analyzer Model 4300 (LI-COR Biosciences, Lincoln, NE, USA).

### 2.5. SSR-Analyses

For PCR amplification, M13 tailing [48] was performed using the SSR markers published in the sunflower reference map [33]. The sequences were obtained from the NCBI database. Published primers were slightly modified or newly derived and a M13-tail (5′-TTTCCCAGTCACGACGTT-3′) was added to the forward primer, which allowed amplification by the M13-IRD800-primer (5′-AGGGTTTTCCCA GTCACGACGTT-3′). For the PCR reaction, 2 μL DNA (50 ng/ μL) was mixed with 13 μL master mix. The master mix contained 0.3 μL dNTP (10 mM), 10.6 μL H_2_O, 1.5 μL 10× PCR buffer with Mg, 0.15 μL M13-IRD800-primer (5 pmol/μL), and 0.15 μL Taq polymerase (5 U/μL), 0.15 μL forward-Primer (5 pmol/μL), and 0.15 μL reverse-Primer (5 pmol/μL). PCR amplification was performed: denaturation 5 min, 95 °C, followed by 36 cycles of 20 s denaturation at 95 °C, 20 s annealing (T_A_ = 55–60 °C, depending on the primer combination), 30 s polymerization at 72 °C. The PCR program was finalized by 5 min extension at 72 °C. SSR markers were separated on denaturing polyacrylamide gels using the DNA Analyzer Model 4300 (LI-COR, Biosciences, Lincoln, NE, USA).

### 2.6. Cloning and Sequencing of AFLP markers

For the cloning of markers (three AFLP markers close to the *Rf-PET2* gene and one AFLP marker next to the *Rf1* gene), PCR reactions were run again with radioactively labeled *Eco*RI primers for the parental lines, RHA 265(PET2) and IH-51, as well as RHA 325 and HA 342. For a better identification of the markers of interest the corresponding fertile and male sterile bulks were also included in these analyses. Cloning and sequencing of the AFLP markers was performed as described in Sajer et al. [49].

### 2.7. Sequence-Tagged Site (STS) Marker Analyses

Primers were derived from the sequences using the internet program Web Primer (http://www.yeastgenome.org/cgi-bin/web-primer). The primer sequences are presented in Table 1. The master mix for the 25 μL STS-PCR reaction was performed as duplex PCR containing 0.5 μL dNTP (10 mM), 5 μL 10× PCR-reaction buffer with MgSO_4_, 0.4 μL Taq Polymerase (5 U/μL), and 11.6 μL H_2_O. The primer mix consisted of 1 μL forward primer, 1 μL reverse primer (10 pmol/µL each) for the marker, as well as 1 μL forward primer, 1 μL reverse primer (10 pmol/µL each) for the internal control (*atp9*, *coxII* or *rbcL*), and 0.2 μL H_2_O. Finally, 3.3 μL template DNA (20 ng/μL) was added. All reactions were performed with the PCR cycler program: 3 min, 94 °C denaturing, followed by 40 cycles (1 min 94 °C, 2 min 52–65 °C (depending on the primers, Table 1), 2 min 72 °C) and finished with 4 min of extension. PCR reactions were separated on 2% agarose gels using a 100 bp ladder (NEB Corporation, Ipswich, MA, USA) as the marker.

### 2.8. Linkage Analyses

Linkage analyses were performed using the Joinmap5 according to the user manual [50]. The maximum likelihood function was applied for mapping. The Haldane function was used to obtain the genetic distances in centiMorgan (cM) [51].

## 3. Results

### 3.1. CMS PET2 Male Sterility and Its Fertility Restoration in the Mapping Population

The male sterility phenotype in CMS PET2 is characterized by medium-sized anthers that produce a very small amount of white pollen, if at all, which is not shed. Artificial pollination using the white pollen resulted in a low degree of seed set. Staining of the white pollen by Alexander’s stain also indicated a varying, low percentage of viable pollen. This showed that the microspore development in the PET2-cytoplasm was either not totally disrupted or only disturbed at a very late stage. Fertility restoration by the line IH-51 resulted in fully restored male fertile F_1_-hybrids showing normal anthers, which produced large amounts of yellow pollen. Pollen viability of the hybrids was estimated to be 98.2% ± 1.2, indicating a sporophytic mode of fertility restoration. Capacity of stable fertility restoration of the line IH-51 has been verified in field trials over several years. The segregating progeny of a cross between the CMS line RHA 265(PET2) and the restorer line IH-51 was evaluated for male fertility/sterility to determine the number of genes involved in fertility restoration. There was a clear distinction between male sterile plants that did not shed pollen and male fertile plants producing pollen. In F_3_, 14 plants of each fertile F_2_-individual were sown to be evaluated for male fertility/sterility to distinguish between F_2_-individuals being homozygous or heterozygous for the restorer gene *Rf-PET2*. Only F_3_ progenies showing no male sterile plants were scored as homozygous fertile. Comparing the expected ratio of 1:2:1 for one gene with the observed segregation ratio of 43 homozygous male fertile: 105 heterozygous fertility restored: 45 homozygous male sterile, *χ*^2^ = 1.536 (degree of freedom = 2, *p* = 0.46) was calculated. As the null hypothesis could not be rejected, the presence of one major restorer gene, which we named *Rf-PET2*, was confirmed.

### 3.2. Mapping the Rf-PET2 Gene to Linkage Group 13

**Figure 1:**
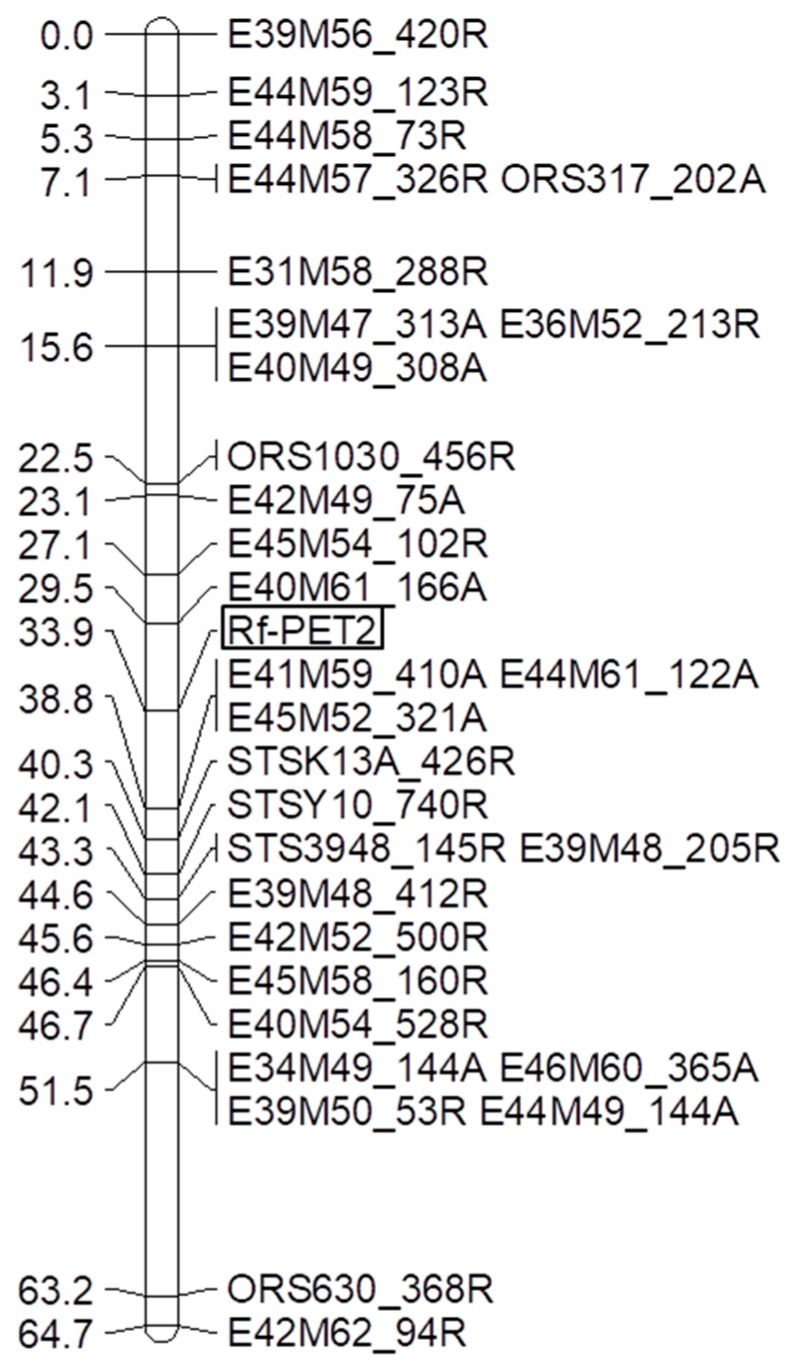
Linkage map for the restorer gene *Rf-PET2*. AFLP markers, SSR markers, and STS markers were integrated. The AFLP markers are given with their primer combination followed by the size of the marker in attraction (A) or in repulsion (R).

Bulked segregant analyses were performed for 256 AFLP primer combinations using two bulks of each seven homozygous male sterile F_2_-plants and two bulks of each seven homozygous male fertile plants of the F_2_-population RHA 265(PET2) × IH-51. In total, 123 polymorphic primer combinations with 1–5 polymorphisms were identified, resulting in 191 polymorphisms. The polymorphic primer combinations were analyzed in the F_2_-population and 146 AFLP markers could be mapped in the segregating population. Due to the high number of markers available only a subset of markers closely linked to the *Rf-PET2* gene were integrated into the map (Figure 1). To assign the *Rf-PET2* gene to a linkage group of the reference genome map three SSR primer combinations ORS317, ORS1030, and ORS630 were also mapped in the F_2_-population RHA 265(PET2) × IH-51. *Rf-PET2* mapped together with 24 AFLP markers, three sequence-tagged site (STS) markers and three SSR markers into one linkage group, LG13 (Figure 1). The map showed a collinear order of the SSR markers in comparison to the general genetic sunflower map [33]. The restorer gene *Rf-PET2* mapped between the ORS1030 markers and ORS630 as the restorer gene *Rf1* for CMS PET1.

### 3.3. Mapping an Additional AFLP Marker to the Rf1 Gene

**Figure 2:**
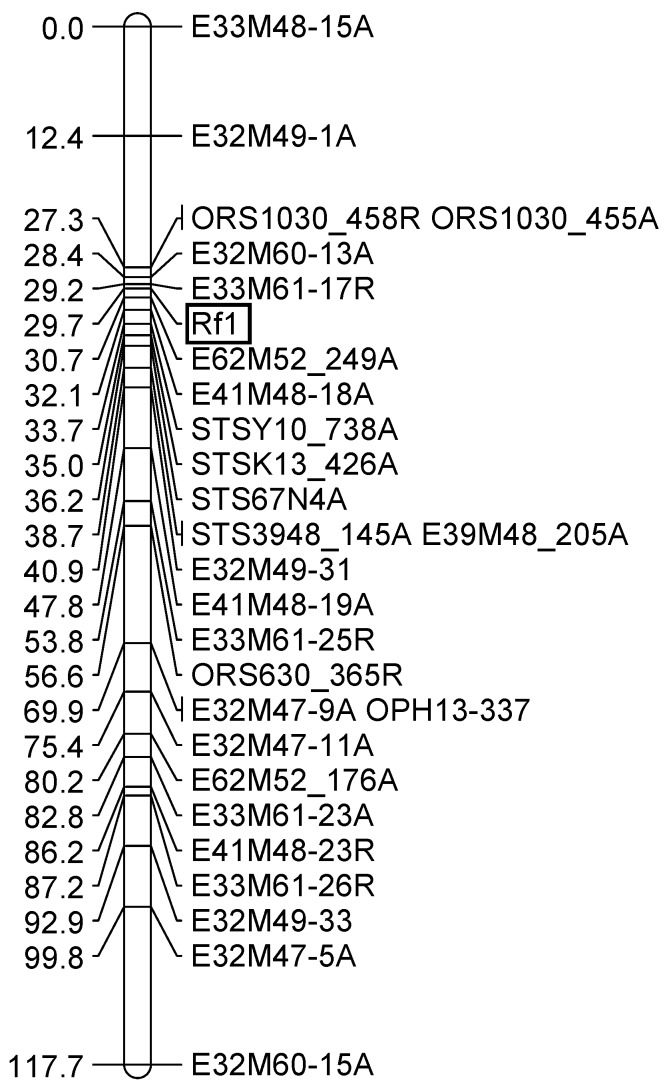
Mapping of new markers into the existing map around the restorer gene *Rf1*. The AFLP markers consist of primer combination, no., and information on attraction (A)/repulsion (R).

For identifying markers more closely linked to the restorer gene *Rf1*, 256 new AFLP primer combinations (E47-E62 combined with M47-M62) were screened in the F_2_-population of the cross RHA 325(PET1) × HA342 by bulked segregant analyses. Two bulks of 10 homozygous male-fertile F_2_-progenies and two bulks of 10 homozygous male sterile F_2_-progenies were compared. In total, 55 polymorphic primer combinations were identified, representing 82 polymorphisms. After mapping all new markers, only the marker E62M52_249A shown in Figure 2 proved to be closer to *Rf1* than previously mapped markers [44]. In addition, a new STS-marker STS67N4 was mapped closely to the restorer gene *Rf1*. This marker was derived from the BAC-end sequences of BAC 67N4, which had originally been identified by hybridization of OP-K13_456 to the BAC library of RHA 325 [36].

### 3.4. Cloning and Sequencing of AFLP Markers Close to Rf-PET2 and Rf1

**Figure 3:**
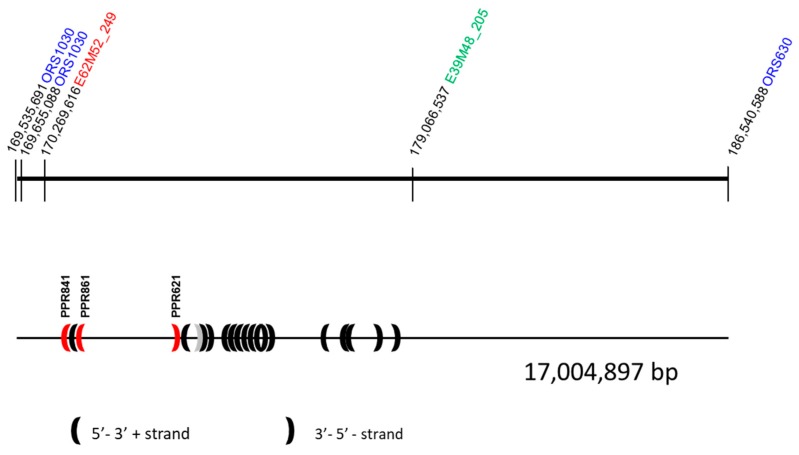
Position of the markers used in mapping of restorer gene *Rf-PET2* according to the BLAST against HanXRQ v1r1 [35]. Potential candidate genes in the area are shown according to Goryunov et al. [37]. The three PPR genes that showed SNPs significantly associated with fertility restoration by *Rf1* are marked in red [36].

AFLP markers can be divided into two classes: (1) AFLP markers smaller than 165 bp that can only be used to develop overgo probes for hybridizations against BAC-filters in a map-based-cloning approach and (2) AFLP markers in the range 200–460 bp that can be used to design overgo probes as well as primers for STS markers. Three AFLP markers close to *Rf-PET2*, E45M52_321A, E39M48_412R, and E39M48_205R, and the newly identified AFLP marker E62M52_249A close to *Rf1* were successfully cloned and sequenced. For three of the markers, E39M48_412R, E39M48_205R, and E62M52_249A, one sequence each is presented, whereas for the eight clones of E45M52_321A a consensus sequence is shown due to observed single nucleotide polymorphisms in the different clones (Supplementary Table S1). Using the Basic Local Alignment Search Tool (BLAST) to find similarities of these sequences against the HanXRQ sunflower genome assembly v1r1 [35] gave the highest homology of E62M52_249 and E39M48_205 (>96%) to LG13, whereas E45M52_321 and E39M48_412 showed lower homologies to LG13 than to other linkage groups (Supplementary Table S1). E62M52_249 and E39M48_205 were positioned between ORS1030 and ORS630, supporting a collinear order of the markers in the genome sequence and confirming the localization in the distal region of LG13 (Figure 3).

STS markers designed for E45M52_321A resulted in a monomorphic banding pattern in the investigated lines RHA 265(PET2), RHA 325(PET1), IH-51, and HA 342, indicating that the polymorphism was restricted to the original restriction sites. A new polymorphic STS-marker STS3948_145 was derived from the AFLP marker E39M48_205R, which could be mapped in both populations. From E62M52_249A, linked to *Rf1*, an overgo probe was designed and used for hybridization to the BAC library of HA 383 identifying the BAC clones 006N12, 447N06, and 480G04 [36].

### 3.5. Comparative Mapping of the Restorer Genes Rf-PET2 and Rf1

**Figure 4:**
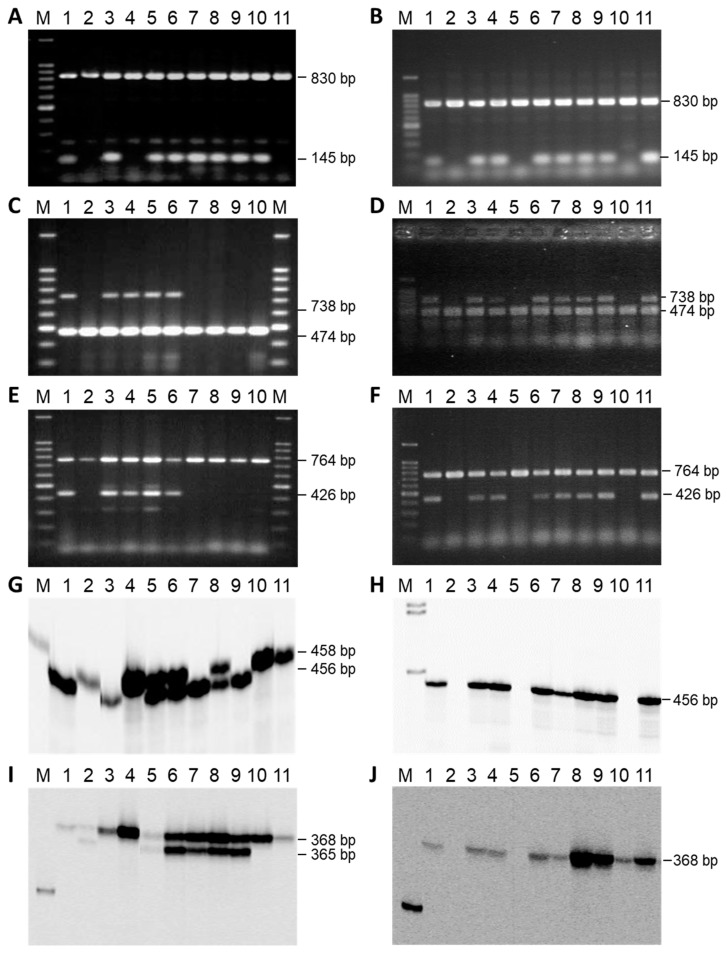
STS markers and SSR markers used for the comparative mapping of the two restorer genes *Rf1* and *Rf-PET2* in RHA 325(PET1) × HA 342 and RHA 265(PET2) × IH-51, respectively. (**A**,**B**) STS3948_145, internal control *rbsL*; (**C**,**D**) STSY10_740, internal control *atp9*; (**E**,**F**) STSK13_426, internal control *coxII*; (**G**,**H**) ORS1030; (**I**,**J**) ORS630; M 100 bp ladder; 1 female parent, 2 male parent, 3–11 F_2_-individuals of the cross combination RHA 325(PET1) × HA 342 (left side) or of the cross combination RHA 265(PET2) × IH-51 (right side), respectively. The phenotypes and genotypes of the F_2_-progenies are given in Supplementary Table S2.

For the comparative mapping, three SSR markers (ORS317, ORS630, and ORS1030) of linkage group 13 of the general genetic sunflower map [33], as well as three STS markers, were analyzed for polymorphisms in the two mapping populations based on the crosses RHA 265(PET2) × IH-51 and RHA 325(PET1) × HA 342 (Figure 4). For *Rf-PET2*, all three SSR markers (ORS317, ORS630, and ORS1030) could be mapped in the population, as amplification products with sizes of 202 bp, 368 bp, and 456 bp, respectively, were only observed in RHA 265(PET2), but not in IH-51. However, for the *Rf1* gene ORS317 did not amplify in RHA 325 and HA 342 at all and ORS630 resulted in two PCR products of 368 bp and 365 bp (Figure 4). One band (368 bp) proved to be monomorphic and only the second band specific for HA 342 was segregating in the population. ORS1030 also resulted in two PCR products of 456 bp (specific for RHA 325) and 458 bp (specific for HA 342), which were mapped as ORS1030_456A and ORS1030_458R (Figure 2). STS markers STSY10_740 and STSK13_426 (also named HRG01 and HRG02 [34]), developed in the cross RHA 325(PET1) × HA 342, could also be mapped in the cross RHA 265(PET2) × IH-51. In addition, the newly developed STS marker STS3948_145A (Figure 4) was mapped in both mapping populations. In general, mapping of the markers in both populations was possible, because RHA 325 and RHA 265 carry the *Rf1* gene, whereas HA 342 and IH-51 do not have it.

Mapping of the *Rf-PET2* gene together with three SSR markers of linkage group 13 demonstrated that this restorer gene for the new CMS source is located on the same linkage group as *Rf1* (Figure 1). The map also showed a collinear order of the SSR markers in comparison to the general genetic sunflower map [33]. STS-K13_426R, STS-Y10_740R, STS3948_145R were mapped with distances of 6.4 cM, 8.2 cM, and 9.4 cM to the *Rf-PET2* gene (Figure 1). In the cross RHA 325(PET1) × HA 342, the markers E62M52_249A, STSY10_740A, STSK13_426A, STS67N4, and STS3948_145A were mapped with 1 cM, 4 cM, 5.3 cM, 6.5 cM, and 9 cM from the *Rf1* gene (Figure 2). Mapping of the STS markers confirmed the close location of *Rf-PET2* and *Rf1*.

## 4. Discussion and Conclusions

*Rf1* had previously been mapped to linkage group 13 of the general sunflower genetic map using the SSR primer combination ORS1030 [34]. Mapping the SSR markers ORS317, ORS630, and ORS1030 of this linkage group in RHA 265(PET2) × IH-51 demonstrated that *Rf-PET2* is also located on the same linkage group 13 as *Rf1*. In the comparative mapping approach, the newly developed markers STS3948_145A, STSY10_740A, and STSK13_426A closely linked to *Rf1* could be also mapped to the restorer gene *Rf-PET2*. This indicates that both restorer genes *Rf-PET2* and *Rf1* are close to each other on the lower part of LG13. Co-localization of different restorer genes has also been observed in other plant species. In rice, five restorer genes (*Rf-1a, Rf-1b*, *Rf4*, *Rf5,* and *Rf6(t)*) responsible for fertility restoration in the presence of three different CMS systems, cms-BT (Boro II), cms-WA (Wild Abortive), and cms-HL (Honglian), were also all located on one linkage group corresponding to chromosome 10 [52,53,54,55,56]. Also, in rye, the restorer genes *Rfg1* and *Rfc4,* and *Rfp1* and *Rfp2*, which allow fertility restoration in the presence of the G-cytoplasm and P-cytoplasm, respectively, were all mapped together on chromosome 4RL [57,58,59].

Several restorers of fertility genes have been isolated in recent years [1], which, apart from a few like the *Rf2* gene in maize, representing an aldehyde dehydrogenase [60,61], belong to the class of PPR-type restorer genes. PPR genes represent a large family of genes (> 450 in *Arabidopsis*) that are characterized by a pentatricopetide motive (35 amino acids) and play a role in processing RNA in mitochondria and chloroplasts [62,63]. The restorer gene in Petunia was the first of these PPR-type restorer genes to be cloned [64]. All other restorer genes of this type have been isolated by a combination of a map-based cloning approach and a candidate gene approach for PPR genes in closed contigs around the restorer genes [53,65,66,67,68]. The characterization of the restorer gene loci has demonstrated that apart from *Brassica* [69,70], the restorer genes are all imbedded in clusters of PPR-type genes. PPR proteins involved in fertility restoration belong to a subgroup within the P subfamily of PPR proteins named Restorer of Fertility Like (RFL) proteins [71]. This group of *PPR* genes shows high evolution rates after the divergence of species, resulting in paralogs that can adapt fast to newly upcoming CMS *orfs* and silence them [71,72]. P-type PPR proteins are involved in RNA processing within the organelles [73]. As *Rf-PET2* reduces the transcript level of the co-transcript of *orf288* and *orf231* in the fertility-restored F_1_-hybrids carrying CMS PET2, its action might correspond to a P-type PPR gene.

According to the AFLP and SSR analyses reported in this paper, the population RHA 265(PET2) × IH-51 is highly polymorphic, which is helpful for a map-based cloning approach of the restorer gene *Rf-PET2*. BLAST of three sequenced AFLP markers mapping close to *Rf-PET2* against HanXRQ v1r1 confirmed the localization between ORS1030 and ORS630. This region contains a large cluster of PPR genes [37]. Mutations of one of these *PPR* genes might have created the *Rf-PET2* gene. Comparison of the genomic sequences of this region between RHA 265, IH-51, RHA 325, and HA 342 should reveal the restorer gene *Rf-PET2*. As the restorer line RHA 265 carrying *Rf1* was used as the maintainer line for CMS PET2, the restorer gene *Rf-PET2* is different from *Rf1*. Cloning and functional analysis of the two restorer genes will help to better understand developmental processes involved in male sterility and fertility restoration.

Our study provided the location of *Rf-PET2* and made STS markers available, which are linked to the restorer gene *Rf-PET2*. These markers can now be used for marker-assisted development of a restorer line pool for the new CMS source PET2. Sunflower represents an interesting crop for the future based on yield predictions for the expected climate change [74]. However, the use of different CMS sources in sunflower hybrid breeding would be advantageous to reduce risks of pathogen attacks and to explore effects of other cytoplasmic traits.

## Figures and Tables

**Table 1:** Primer sequences used for the comparative mapping.

Marker	Size bp	Forward Primer	Reverse Primer	T_A_	Genbank Accession
ORS317 *	200	TTTCCCAGTCACGACGTT CGTATGCTTAATTCTTTCTCT	TTTGGCAGTTTGGTGGCTTA	55	BV006839
ORS1030 *	426	TTTCCCAGTCACGACGTT TGATGTAGTTAAGGAAGTTGTG	CGATCAATTTATATGACCGAATTACC	55	BV006414
ORS630 *	344	TTTCCCAGTCACGACGTT CGACCCGGATATGTAAC	TGTGCTGAGGATGATATGCAG	55	BV006710.1
STSY10_740	738	AAACGTGGGAGAGAGGTGG	AAACGTGGGCTGAAGAACTA	65	[44]
STSK13_426	426	TATGCATAATTAGTTATACCC	ACATAAGGATTATGTACGGG	60	[44]
STS3948_145	145	GTTTTTGGGACATCGCCATTTT	GCGGGTGGAAATCCATATATGAG	52	This study
STS67N4	471	TTTGAGGGCTCATCTCCAGTCA	TGCAATGAGCTTAGCCCATCG		This study
*coxII*	764	CGAGAAATAGATGCTCAGCCTG	GATAATGCGCAGTGGAAAGG	60	X62341
*atp9*	474	GGTGCAAAATCAATAGGGGCCG	ACCGAATGAATGCGTCACAAGG	65	X51895
*rbcL*	830	CGTCTGGAAGATTTGCGAATC	GGGTGCCCTAAAGTTCCTCC	52	AF097517

* M13 sequence is underlined.

## Supplementary Materials

A supplementary file is available at: https://www.mdpi.com/2073-4425/11/3/269/s1. Table S1: List of sequenced AFLP markers, including their sequence and their location in HanXRQ genome assembly v1r1 [35]. Table S2: Phenotype and genotype of the loaded PCR-products in Figure 4.
