# Supplementary material for: Mapping of the New Fertility Restorer Gene Rf-PET2 Close to Rf1 on Linkage Group 13 in Sunflower (Helianthus annuus L.)
Source: Genes (Basel). 2020 Mar 1;11(3):269. doi: 10.3390/genes11030269 (PMC7140827; doi:10.3390/genes11030269)
Supplement: Supplementary file 1 [file genes-11-00269-s001.pdf]

Supplementary Table S1: List of sequenced AFLP-markers, including their sequence and their location in HanXRQ genome assembly v1r1 [35]. AFLP-primer sequences are shown in green. The obtained sequence length including the primer sequences is given in brackets. Homologies are shown till the first hit on Chr13.

> E45M52_321A, attraction(320 bp)_*Rf-PET2*
GACTGCGTACCAATTCATG
GGAGGCTGTAGGAGGTGCATAGGCCYGCTCTRAAGAAAGCCAAACCTCACGAGAGTATTTATCATAAACATG
CTGGTAGGAATCTTCAKACACAGTGGTTATGATTAGGGTTCGGATGTGAGCATCGTTTGAGATCCACAGGGT
GTAACTGKGGTTATYAGTGGTGTTGGCTCCAATGGTAACCTTTTGTTTAGGACAGGGTRTTGTTCCATCAAY
ATARCCAAAGAGATTGTGTGATGTAAGAARCAATTCAATCATGAAYYGCCAATATCCATAGTTTTT
GGGTTACTCAGGACTCATC

sseqid	qend	sstart	send	stitle	pcq	pcs	evalue	pident
HanXRQChr17	282	165472997	165473278	len=214723238 previd=17	100	0	4.87e-122	93.617
HanXRQChr03	282	157403227	157402946	len=168485022 previd=03	100	0	4.87e-122	93.617
HanXRQChr01	282	88432518	88432799	len=153905722 previd=01	100	0	2.27e-120	93.262
HanXRQChr14	282	45059888	45060169	len=174509413 previd=14	100	0	1.05e-118	92.908
HanXRQChr14	282	555060	555341	len=174509413 previd=14	100	0	5.01e-102	89.362
HanXRQChr13	280	177825704	177825971	len=197258317 previd=13	95	0	5.46e-42	77.323

>E39M48-205R, repulsion (205 bp)_*Rf-PET2*
GACTGCGTACCAATTCAGA
TCAAAACTAGTTGTTTTTGGGACATCGCCATTTTTTTGTCGAGAACTTTAGTTTTCGTGTTTTCTATTCTTT
TATAATAGTTAGTGTGATATATATGAGGGGTTATTTTCAGGACATCGTCGAGTTTTGTCCACCTCATATATG
GATTTCCACCCGCCATGAACC
GTGTGTTACTCAGGACTCATC

sseqid	qstart	qend	sstart	send	stitle	pcq	pcs	evalue	pident
HanXRQChr13	1	165	179066537	179066375	len=197258317 previd=13	100	0	1.32e-70	96.364

>E39M48-412R, repulsion (395 bp)_*Rf-PET2*
GACTGCGTACCAATTCAGA
ATAGAGATCCTGGTGTTTTGGTTCGAGTCGGTTCGGCCTTGGGCCGTGATGGTGATCTTTTGGGAAGTCATG
GGCCGTCTTCCGTTGATCATGCAGTTTTGGAAAACTACGGCCGCGTTGCCGAAGATGAAACCAACGGTCCCG
TAAATGTAGCATTCTTTGTAGAATTGTCGTTGTGAGTGGACGTAGAGGGTGTCTTGATATTCTTCAAAACCG
CAATGGTAGAAGACCGATAAGTCGGACCCGGACCGTAGAGCTACGGCTTGGTGGGTTTTGTGGCCCGCGGT
GTTGCGGAAGGTTATGCCACGAGCAATGAATCCGGACCCGATTACAACTGTTGTTAGATGGAAACAAAC
GTGTTACTCAGGACTCATC

sseqid	qstart	qend	sstart	send	stitle	pcq	pcs	evalue	pident
HanXRQChr07	1	356	100451019	100450665	len=103871911 previd=07	99	0	2.1e-176	98.315
HanXRQChr07	1	356	99627268	99627622	len=103871911 previd=07	99	0	2.15e-161	95.787
HanXRQChr07	1	356	100366548	100366194	len=103871911 previd=07	99	0	4.65e-158	95.225
HanXRQChr07	1	356	100397444	100397090	len=103871911 previd=07	99	0	2.16e-156	94.944
HanXRQChr07	1	219	100420873	100420655	len=103871911 previd=07	61	0	3.91e-99	96.804
HanXRQChr07	257	356	100412503	100412405	len=103871911 previd=07	28	0	4.26e-39	97
HanXRQChr13	92	322	183457595	183457366	len=197258317 previd=13	64	0	7.12e-37	79.31

>E62M52_249A, attraction (250 bp)_*Rf1*

GACTGCGTACCAATTCCTT
AGCTGAATCCATGTGGGGTATGGAAAAAACAACTTGAATGCAAGGAAGACGACACATGCTACTTCATGGAAC
GTATCTGGGTTCCATCCTATGGAAACCTACGAGAGCTTGTGATGGACGAAGCACACAAGTCTAGTTACTCGG
TGCATACTGGTGGCCCCACATGAAAGCTAACATAGTGACCTATGTTAGTAAATGCTTGACCTGTGCTA
GGGTTACTCAGGACTCATC

sseqid	qstart	qend	sstart	send	stitle	pcq	pcs	evalue	pident
HanXRQChr13	1	155	170269616	170269770	len=197258317 previd=13	73	0	1.78e-65	96.129

Supplementary Table S2: Phenotype and genotype of the loaded PCR-products in Figure 4

	1	2	3	4	5	6	7	8	9	10	11
**A** RHA325(PET1) x HA342, STS3948_145R, in attraction	RHA325 (PET1), F, Rf1Rf1	HA342 F, rf1rf1	5200/95 15 F, Rf1Rf1	5200/95 16 S, rf1rf1	5200/95 17 F, rf1Rf1	5200/95 18 F, rf1Rf1	5200/95 19 F, Rf1Rf1	5200/95 20 F, rf1Rf1	5200/95 21 F, Rf1Rf1	5200/95 22 S, rf1rf1	5200/95 23 S, rf1rf1
**B** RHA265(PET2) x IH-51, STS3948_145R, in repulsion*	RHA265 (PET2) S, rr	IH-51 F, RR	1 F, rR	2 S, rr	3 F, RR	4 S, rr	5 F, rR	6 F, rR	7 S, rr	8 F, RR	9 S, rr
**C** RHA325(PET1) x HA342, STSY10_740, in attraction	RHA325 (PET1), F, Rf1Rf1	HA342 F, rf1rf1	5200/95 104 F, rf1Rf1	5200/95 105 F, Rf1Rf1	5200/95 106 F, rf1Rf1	5200/95 110 F, rf1Rf1	5200/95 101 F, rf1Rf1	5200/95 103 F, Rf1Rf1	5200/95 118 S, rf1rf1	5200/95 119 S, rf1rf1	-
**D** RHA265(PET2) x IH-51, STSY10_740, in repulsion*	RHA265 (PET2) S, rr	IH-51 F, RR	1 F, rR	2 S, rr	3 F, RR	4 S, rr	5 F, rR	6 F, rR	7 S, rr	8 F, RR	9 S, rr
**E** RHA325(PET1) x HA342, STSK13_426, in attraction	RHA325 (PET1), F, Rf1Rf1	HA342 F, rf1rf1	5200/95 106 F, rf1Rf1	5200/95 110 F, rf1Rf1	5200/95 111 F, rf1Rf1	5200/95 112 F, rf1Rf1	5200/95 119 S, rf1rf1	5200/95 126 S, rf1rf1	5200/95 130 S, rf1rf1	5200/95 142 S, rf1rf1	-
**F** RHA265(PET2) x IH-51, STSK13_426, in repulsion*	RHA265 (PET2) S, rr	IH-51 F, RR	1 F, rR	2 S, rr	3 F, RR	4 S, rr	5 F, rR	6 F, rR	7 S, rr	8 F, RR	9 S, rr
**G** RHA325(PET1) x HA342, ORS1030, in attraction	RHA325 (PET1), F, Rf1Rf1	HA342 F, rf1rf1	5200/95 15 F, Rf1Rf1	5200/95 16 S, rf1rf1	5200/95 17 F, rf1Rf1	5200/95 18 F, rf1Rf1	5200/95 19 F, Rf1Rf1	5200/95 20 F, rf1Rf1	5200/95 21 F, Rf1Rf1	5200/95 22 S, rf1rf1	5200/95 23 S, rf1rf1
**H** RHA265(PET2) x IH-51, ORS1030, in repulsion*	RHA265 (PET2) S, rr	IH-51 F, RR	8 F, RR	14 F, RR	21 F, rR	26 F, RR	30 F, RR	62 F, RR	68 F, RR	77 F, rR	86 S, rr
**I** RHA325(PET1) x HA342, ORS630, in repulsion	RHA325 (PET1), F, Rf1Rf1	HA342 F, rf1rf1	5200/95 79 F, Rf1Rf1	5200/95 80 F, Rf1Rf1	5200/95 83 F, rf1Rf1	5200/95 89 S, rf1rf1	5200/95 90 F, rf1Rf1	5200/95 91 F, rf1Rf1	5200/95 92 S, rf1rf1	5200/95 93 F, Rf1Rf1	5200/95 94 F, Rf1Rf1
**J** RHA265(PET2) x IH-51, ORS630, in repulsion*	RHA265 (PET2) S, rr	IH-51 F, RR	1 F, rR	2 S, rr	3 F, RR	4 S, rr	5 F, rR	6 F, rR	7 S, rr	8 F, RR	9 S, rr

* R and r stand abbreviated for the dominant Rf-PET2 and the recessive rf-PET2 allele, respectively
